# Decompression of peripheral nerve trunks in leprosy to prevent the development and progression of deformities

**DOI:** 10.4103/0019-5413.38586

**Published:** 2008

**Authors:** Sajid Husain, Birjendra Mishra

**Affiliations:** Department of Plastic and Reconstructive Surgery, National JALMA Institute for Leprosy, Taj Ganj, Agra, India; 1 Department M, NJIL, Agra; Department of SPM, Gandhi Medical College, Bhopal, India

**Keywords:** Deformity, leprosy, peripheral nerve decompression

## Abstract

**Background:**

Peripheral nerve involvement results in deformities in leprosy. High doses (40-60 mg) of steroids along with anti-leprosy drugs is the preferred treatment, even though 70-75% cases still develop deformity. Early surgical decompression of nerves gives better chances of preventing deformity. We have analyzed the role of early surgical decompression in such cases.

**Materials and Methods:**

Five hundred nerves (386 ulnar, 60 median and 54 posterior tibial) not responding to the medical treatment in 12 weeks, were undertaken for external and internal nerve trunk decompression. These cases were followed up for five to 20 years at various intervals.

**Results:**

The pain in nerve (neuralgia) recovered in all cases of ulnar, median and posterior tibial nerves. Full sensory recovery to pinprick and feather or cotton wool touch was seen in 50% cases of all the three nerves. Twenty percent cases maintained the preoperative levels of sensory status. Plantar ulcers healed within six months after decompression of posterior tibial nerve but six cases showed recurrence. Overall motor recovery in ulnar nerve was 89% and 70% in median nerve.

**Conclusions:**

The sensory recovery restores protective sensation which prevents secondary injuries. The improvement of motor power gives better function and improves the appearance, which in the absence of surgical intervention was not possible.

## INTRODUCTION

Leprosy is a disease of nerves and known for its deformities. The peripheral nerve involvement in leprosy is common and results in damage leading to various deformities. The commonly involved nerves in the upper limb are the ulnar and median and in the lower limbs - posterior tibial and lateral poplitial nerve in that order.^1^

Nerves are known to get entrapped at various anatomical sites clinically manifesting in paresthesia and paresis even in nonleprotic conditions. It is also known that inflamed swollen nerves due to any cause are more prone to entrapment.^2^–^6^

In multi bacillary (MB) leprosy, nerve thickening occurs following invasion of bacilli in nerve tissue or following lepra reactions in the nerve, while in paucibacillary (PB) leprosy, hypersensitivity reaction leads to sudden inflammation. Swollen edematous nerves passing through tunnel-like structures suffer by getting compressed (entrapment neuropathy). Sensory loss is the most commonly reported symptom followed by motor loss. The autonomic function loss leads to dryness, fissures etc. Motor loss manifests in the form of paresis or paralysis in different parts (claw hand, foot drop, lagophthalmos, etc.); sensory loss manifests in the form of burn blisters, trophic ulcers. In India 25-30% of leprosy cases develop deformities.^7^

Several structures can compress the nerve and cause ischemia, venous obstruction, capillary stasis, intrafunicular hypoxia, edema and increased intrafunicular tension. The net result of entrapment/ischemia is initial slowing of conduction velocity and later full conduction block and ultimately paralysis. The diseased and thickened ulnar nerve can get damaged further due to physical trauma related to the above mentioned anatomical factors.

Median nerve involvement is infrequently seen in leprosy but whenever it gets damaged, it results in functional loss affecting the pinch and grasp functions. Clinically, the median nerve involvement commonly presents as carpal tunnel syndrome, the wrist being the usual site for its involvement in leprosy. Muir^8^ was of the opinion that the painful nerve causing progressive paralysis is suitable for decompression accomplished by splitting of nerve sheath that will conserve its function.^4^,^6^

Ulceration of the foot is due to sensory loss in the sole consequent upon the involvement and damage to the posterior tibial nerve. The inflamed nerve is usually entrapped and compressed in the tarsal tunnel behind the medial malleolus. Steroid therapy to treat the inflammation is insufficient and entrapment needs surgical decompression.^2^,^9^,^10^

Early detection and diagnosis of nerve damage can be done by regular and periodic nerve function assessment (sensory by pin prick and cotton wool/feather touch; motor status by Medical Research Council (MRC) muscle power grading) of all susceptible nerves on every visit. If the patient is developing nerve damage, steroids must be started in adequate doses; 60 mg daily for first fortnight followed by a tapering of 5 mg every two weeks is the protocol of choice. If there is no improvement in eight to 12 weeks as evident by decrease in pain and tenderness over the nerve, reduced paresthesia or if one observes worsening/deterioration in motor power, then such cases should be considered for nerve decompression.^5^,^6^

Surgical intervention in the form of epineurotomy by multiple longitudinal incisions and external decompression to relieve the internal pressure throughout the involved segment is considered the treatment of choice after failure of steroids.

The present study is based on research hypothesis that the cases not responding to steroid therapy if treated with surgical decompression at an early stage, have better chances of preventing further nerve damage and occurrence of deformity as compared to those who continued with steroid therapy only.

## MATERIALS AND METHODS

During 1982 to 2002, 386 ulnar nerves, 60 median nerves and 54 posterior tibial nerves were undertaken for decompression. These cases were on 40-60 mg of steroids for more than 12 weeks and did not show any improvement. The cases were paucibacillary, multibacillary and neuritic type. The paucibacillary cases were having less than five anesthetic patches along with nerve involvement while the multibacillary cases were having eight to fifteen anesthetic patches with skin infiltration and nerve involvement. The neuritic cases were not having any anesthetic patch over the skin; only thickened painful nerve with paresthesia was seen.

All these cases were of less than six months' duration and on anti-leprosy treatment. A detailed history was recorded from each patient. This included the duration of disease, duration of neural symptoms, history of treatment, mainly the anti-leprosy drugs and steroids with doses and duration of steroid intake.

### Ulnar nerve:

Three hundred and eighty-six cases were followed up for variable periods (5-20 years) following the decompression. Out of these, 349 cases were male and 37 were female. The youngest patient was nine years old while the oldest was 76 years, the majority being adults (mean age was 32 years). Of 386 cases, 228 cases were PB type, 112 MB type, and 46 neuritic (N) type of leprosy. Only 152 patients complained of severe nerve pain at the elbow which woke them up during nights.

Detailed clinical examination was carried out. The ulnar nerve was palpated for thickening, tenderness and presence of abscess. The sensory function was examined with pinprick and feather touch/cotton wool. Complete charting of affected muscles and their motor power on MRC grading was done.

### Median nerve:

Sixty patients had nerve decompression (49 male and 11 female). The age varied from 14 to 75 years (mean age 28.5 years). Thirty-three cases were PB, 18 MB and nine cases were N type. All these had history of pain in lower forearm and wrist region and sensory loss over the palmar area of the thumb, index and middle fingers (not able to feel or differentiate pinprick, feather touch/cotton wool sensation). Thirty-nine cases had motor power 3 and 21 cases had motor power 1 at the time of decompression.

#### Posterior tibial nerve:

Fifty-four patients having plantar ulcer (innervated by one or more of all three branches) for not more than six months duration were included in this study. Thirty-one cases had history of recurrent nerve pain below the malleolus and the nerve was tender on mild touch. The age varied between 17 and 50 years (mean age 35.5 years), 41 were males and 13 females. The ulcers were superficial. The common sites of ulcers were below the head of the first and second metatarsal (*n* = 29) and heel (*n* = 25). The size of ulcer varied from 1.5 to 3.0 cm. No bony involvement was detected on X-rays.

All the 500 patients were free from diabetes and any other neurological problems and were not able to feel the pinprick/feather touch sensation. These patients were on anti-leprosy treatment along with 40-60 mg/day steroid therapy for more than 12 weeks. There was no improvement in nerve pain and paresthesia was increasing. At this stage these cases were undertaken for nerve decompression. X-ray of chest, routine blood and urine examinations were done before surgery.

##### Surgical procedure

All the nerve decompressions were carried out under local anesthesia without any tourniquet. Lignocaine 2% with adrenaline (1 in 100,000) was used for infiltration anesthesia. The whole surgical procedure was over in 30 minutes.

The ulnar nerve at the elbow was exposed through a longitudinal incision about 7-10 cm above and 3-4 cm below the epicondyle. The deep fascia of the anterior-medial compartment of the upper arm was exposed and de-roofing of the fibro-osseous tunnel was done by cutting the over lying fibrous tissue. The fibrous arch between the two heads of the flexor carpi ulnaris was cut and the distal end of the tunnel was widened. The entire segment of the exposed nerve was cleared from the surrounding adhesions without lifting the nerve from its bed. This resulted in the complete external release of the ulnar nerve. The intraneural decompression was done by medial longitudinal epineurotomy without damaging the vascular network. In 49 cases medial epicondylectomy was also done (because the nerve was subluxating over the epicondyle and getting traumatic neuritis during the elbow movement).

The median nerve was exposed 2 cm above the proximal wrist crease and the adhesions were separated from the surrounding tissues. The flexor retinaculum was cut to its free edge up to the origin of the abductor pollcis brevis fibers. The adhesions inside the carpal tunnel if any, were surgically released. An epineurotomy was done along the length of the nerve taking care not to injure the blood vessels. The maximum thickening of the nerve was noticed just above the proximal edge of the flexor retinaculum.

The procedure used to decompress the posterior tibial nerve is well described by Bourrel (1970).^11^ The nerve was exposed by an “L”-shaped incision just behind the medial malleolus extending 5 to 6 cm above and below it to reach up to the lower border of the calcaneum. The flexor retinaculum was incised and the neurovascular bundle was identified. The nerve was carefully separated from the posterior tibial vessels. Distally thickened inferior calcaneal bands were also incised. The epineurotomy was done on the exposed nerve.^2^ A strip of flexor retinaculum, 5-8 mm wide, was excised. The incision was closed with 3-0 prolene. The weight-bearing was restricted for two weeks.

All the nerves were thickened, edematous and had good vascularity. The adhesions were seen along the whole exposed tract of the nerve.

### RESULTS

All the 386 ulnar nerves decompressed and followed up at five to 20 years had no pain in the ulnar nerve at elbow. The patients allowed touch or pressure on the nerve. Sensory recovery was noted in about 50% of the cases [Table 1]. In 85 patients the ability to feel the touch (subjective sensory improvement) was noticed as early as four weeks, though the usual recovery to pinprick and feather touch too started on an average in about 24 weeks. The improvement gradually progressed to complete recovery and maximum benefits were observed at the end of the first year after nerve decompression.

**Table 1:** Sensory results of decompression of ulnar nerve

Type of disease	No. of cases	Sensory recovery (%) (pinprick and cotton wool/feather touch)
PB	228	90 (39.47)
MB	112	62 (55.35)
N	46	36 (78.26)
Total	386	188 (48.70)

The improvement in motor function was slow and first seen at about 24 weeks in the proximal muscles (flexor carpi ulnaris and flexor digitorum profundus). It was more gradual and in some cases it took about 50 weeks to obtain maximum recovery. The motor recovery in relation to the preoperative MRC grading in different types of leprosy patients is shown in Table 2.

**Table 2:** Results of Motor functions of the ulnar nerve

Total number of cases	Motor power		
	
	Preoperative MRC grade	Postoperative MRC grade	No. of cases
78	Grade - 1	Grade 3	64
		Remained same	14
216	Grade > 1-3	Grade-5	110
		Remained same	86
		Deteriorated to	20
		Grade 0	
92	Grade > 3-5	Grade - 5	86
		Deteriorated to	6
		Grade - 0	

We noted that the ulnar supplied muscles retain their functional ability to prevent the development of deformity up to MRC Grade 3. Hence we grouped the muscle strength into three viz. improved, remained same with usual muscle power and deteriorated, for our postoperative evaluations.

It was observed that out of 386 nerves decompressed the majority (346) were able to retain useful muscle function. Only 26 cases deteriorated while 14 cases retained the same motor status. It was also noted that the cases with motor power 3 or more (MRC grade) for six months or less are more likely to recover whereas others did not improve [Table 2].

In median nerve, full sensory recovery for pinprick and feather touch was seen in 27 cases. While the other 33 cases were able to feel pinprick sensation but cotton wool and feather touch sensation was poor. In 18 cases, motor power improved from Grade 3 to Grade 5 and in nine cases it improved from Grade 1 to Grade 3. Fifteen cases remained the same (Grade 3) postoperatively. All these cases had full functional hand. Twelve cases retained Grade 1 motor power and six cases deteriorated to Grade 0 after surgery and developed deformities [Table 3].

**Table 3:** Results of decompression of median nerve

Total number of cases	Preoperative MRC grade	Postoperative MRC grade	No. of cases
39	Grade 3	Grade 5	18
		Grade 3	15
		Grade 1	-
		Grade 0	6
21	Grade 1	Grade 5	-
		Grade 3	9
		Grade 1	12
		Grade 0	-

For posterior tibial nerve the results of decompression were very promising. The subjective sensory recovery (the patient perception or feel for touch) took place two to three weeks postoperatively while recovery of pinprick and cotton wool/feather touch sensations took place after six to eight weeks in all cases. Twenty-four feet showed recovery of sensation in full sole whereas 16 feet showed recovery in fore-foot, six in mid-foot and eight in heel only. The plantar ulcers healed in all the feet within six months after surgery but recurrence were seen in seven cases though all the cases were advised to wear micro cellular rubber (MCR) chappals.

The results were evaluated on the basis of subjective improvement and objective findings relating to both sensory and motor modalities and periodic comparisons were made with previous assessment values. For ulnar and median nerve, the full sensory recovery (perception to pinprick and feather/cotton wool for touch) was 48%. Relief from pain (i.e patient did not complain of any pain at elbow and wrist, allowed to touch or press the ulnar and median nerves) was 100% after decompression. The motor recovery was seen to Grade 5 in 196 cases. One hundred and fifty cases were able to maintain Grade 3 power in the ulnar nerve. Eighteen cases improved to Grade 5 while 24 cases maintained Grade 3 power in the median nerve. This helped to retain the acceptable muscle function to enable them to lead a fruitful and socially acceptable life by not developing the hand deformities [Table 4].

**Table 4:** Decompression results at glance

	Improved	Remained same	Deteriorated
Ulnar nerve	346 (89.63%)	14 (3.62%)	26 (6.73%)
*n* = 386	(MRC Grade 3-5)	(MRC Grade + 1)	(MRC Grade ‘0’)
Median	42 (70%)	12 20%	6 (10%)
nerve *n* = 60	(MRC Grade 3-5)	(MRC Grade + 1)	(MRC Grade ‘0’)

Posterior tibial nerve decompression helps in improvement of sensations as well as vascularity of foot thereby helping in healing ulcers and preventing further recurrence.^2^,^9^,^10^

### DISCUSSION

The follow-up period varied from 5 to 20 years. Pain was the first symptom to disappear.^3^,^9^ The sensory improvement was noticed in some cases as early as four weeks, though the actual recovery took place in about 20 weeks postoperatively. The improvement gradually progressed to complete recovery and the maximum benefit was noticed in about a year after nerve decompression.^3^ Forty-eight per cent cases showed complete sensory recovery while others had improved sensations as compared to preoperative state. Fifty per cent cases retained their motor power at Grade 5 while 38% cases were able to maintain motor power up to Grade 3. The improvement in motor function was slow to occur and was seen after 24 weeks. It was more gradual and in some cases it took about two years to obtain the maximum motor recovery.

Full sensory recovery was seen in 55% of median nerve cases while the rest of the cases showed partial sensory improvement which helps the patients to be safe from secondary problems like burns, injuries, etc.^4^,^5^,^6^ Thirty-five per cent cases improved to motor power Grade 5 and had normal functional hand while the another 35% were able to maintain motor power Grade 3, with a reasonably good functional hand.

Posterior tibial nerve decompression results were very promising. Forty-four per cent cases obtained full sensory recovery in the sole while 66% cases recovered the sensations partially. The healing of ulcers was also 100% in our series.

In reviewing the literature several authors have reported improvement of sensory status and healing of ulcers with operative decompression of the posterior tibial neurovascular complex. Droogenbroeck^11^ reported healing of ulcers in 75% of cases. Only deep ulcers with bone involvement did not heal. The sensory recovery to pinprick/cotton wool was noted in all the cases which had been operated within six months of neural symptoms. This is also seen in our series. Since in all the groups the number of cases is less than 40 so the neural outcome is at various part of sole was not statistically calculated.

### CONCLUSION

The overall observation suggests that along with basic care of hands and feet, the cases not responding to steroid therapy of 12 weeks, who had nerve decompression showed better functional hands and feet which would not have been possible without timely surgical intervention.
